# Isolation and Taxonomic Identity of Bacteriocin-Producing Lactic Acid Bacteria from Retail Foods and Animal Sources

**DOI:** 10.3390/microorganisms3010080

**Published:** 2015-03-19

**Authors:** Chris Henning, Paul Vijayakumar, Raj Adhikari, Badrinath Jagannathan, Dhiraj Gautam, Peter M. Muriana

**Affiliations:** 1 Department of Animal Science, Oklahoma State University, Monroe Street, Stillwater, OK 74078, USA; E-Mails: cdhenni@okstate.edu (C.H.); paulpv@okstate.edu (P.V.); rajba@okstate.edu (R.A.); vengara@okstate.edu (B.J.); 2 Robert M. Kerr Food & Agricultural Products Centre, Oklahoma State University, 109 FAPC Building, Monroe Street, Stillwater, OK 74078-6055, USA; E-Mail: dhiraj.gautam@okstate.edu

**Keywords:** lactic acid bacteria, bacteriocin, *Listeria monocytogenes*, food preservative

## Abstract

Bacteriocin-producing (Bac^+^) lactic acid bacteria (LAB) were isolated from a variety of food products and animal sources. Samples were enriched in de Man, Rogosa, and Sharpe (MRS) Lactocilli broth and plated onto MRS agar plates using a “sandwich overlay” technique. Inhibitory activity was detected by the “deferred antagonism” indicator overlay method using *Listeria monocytogenes* as the primary indicator organism. Antimicrobial activity against *L. monocytogenes* was detected by 41 isolates obtained from 23 of 170 food samples (14%) and 11 of 110 samples from animal sources (10%) tested. Isolated Bac^+^ LAB included *Lactococcus lactis*, *Lactobacillus curvatus*, *Carnobacterium maltaromaticum*, *Leuconostoc mesenteroides*, and *Pediococcus acidilactici*, as well as *Enterococcus faecium*, *Enterococcus faecalis*, *Enterococcus hirae*, and *Enterococcus thailandicus*. In addition to these, two Gram-negative bacteria were isolated (*Serratia plymuthica*, and *Serratia ficaria*) that demonstrated inhibitory activity against *L. monocytogenes*, *Staphylococcus aureus*, *and Enterococcus faecalis* (*S. ficaria* additionally showed activity against *Salmonella* Typhimurium). These data continue to demonstrate that despite more than a decade of antimicrobial interventions on meats and produce, a wide variety of food products still contain Bac^+^ microbiota that are likely eaten by consumers and may have application as natural food preservatives.

## 1. Introduction

Lactic acid bacteria (LAB) occur naturally on many retail foods and are often added as starter cultures in fermented products widely sold in the marketplace, such as cheese, yogurt, and buttermilk, while others are simply added as bacterial adjuncts, such as acidophilus milk or *Bifidus* yogurt [1,2]. Some of these organisms have gained notoriety for demonstrating a “probiotic” effect when ingested [3], while others are known for producing a variety of antimicrobials including lactic acid, hydrogen peroxide, bacteriophage, and bacteriocins which may be present in foods that are manufactured with them.

Bacteriocins are gene-encoded inhibitory proteins and those produced by Gram-positive LAB are inhibitory mainly to other Gram-positive bacteria. Some bacteriocins even display antagonistic activity towards Gram-positive foodborne pathogens and spoilage organisms [4,5]. Bacteriocins have varying inhibitory spectra and these properties allow for the development of applications towards roles in food safety or preventing spoilage [4,6]. They can also be used in tandem with other antimicrobial treatments (*i.e.*, the hurdle effect) in order to enhance the preservation of food [7]. For example, the antimicrobial effect of nisin is enhanced when mixed with a metal chelating molecule like ethylenediaminetetraacetic acid (EDTA) or a pulsed-electric field which can increase its effectiveness towards Gram-negative bacteria [7]. Considering consumer’s growing interest in natural products and the high cost of foodborne illness, food manufacturers are always looking for new ways to preserve food. The use of bacteriocins as natural antimicrobials may be one possible way to replace or enhance chemical preservatives [3,5].

Lactic acid bacteria may be isolated from many sources as they are widely present in the environment and readily found on the surface of many raw food products and therefore are also found as surface environmental contaminants during food processing and consequently, even on processed foods. They are also present in the intestinal tracts of humans and animals and can therefore be found in feces which is generally considered one vehicle by which they are distributed onto field-grown crops and vegetables. During this study, we examined a variety of food and animal sources, screening for bacteriocin-producing lactic acid bacteria using *Listeria monocytogenes* as a primary indicator organism. Our intention was to identify bacteriocinogenic LAB that might have inhibitory activity against Gram-positive pathogens that could be applicable as food safety adjuncts for use as food preservatives.

## 2. Experimental Section

### 2.1. Bacterial Cultures, Growth Conditions, and Storage

De Man, Rogosa, and Sharpe Lactobacilli (MRS) broth (Difco Laboratories, Detroit, MI, USA) and agar were used to culture bacteriocin-producing (Bac^+^) lactic acid bacteria (LAB) when originally isolated from retail food or animal sources or when retrieved from our laboratory culture collection. Cultures were incubated overnight (12–16 h) at 30 °C by inoculating from an isolated colony, patch-plate smudge from an isolated colony, or a 1% inoculum from pure culture stocks or culture broths. *L. monocytogenes* 39-2 and *Staphylococcus aureus* ATCC 12600 were used as primary and secondary indicators for Bac^+^ organisms, respectively, and were cultured in brain heart infusion media (BHI, Difco) [5,8]. Some Bac^+^ strains were also tested for activity against *E. coli* O157:H7 ATCC 43890, *Enterococcus faecalis* ATCC 19433, and *Salmonella* Typhimurium H3380 that were cultured in tryptic soy broth (TS, Difco) before use. Master cultures were maintained by resuspending cell pellets in milk-based freezing media (11% non-fat dry milk powder, 1% glucose, 0.2% yeast extract) after centrifugation and stored frozen at −80 °C.

### 2.2. Isolation of Bacteriocin-Producing (Bac^+^) LAB from Food and Animal Sources

**Figure 1:**
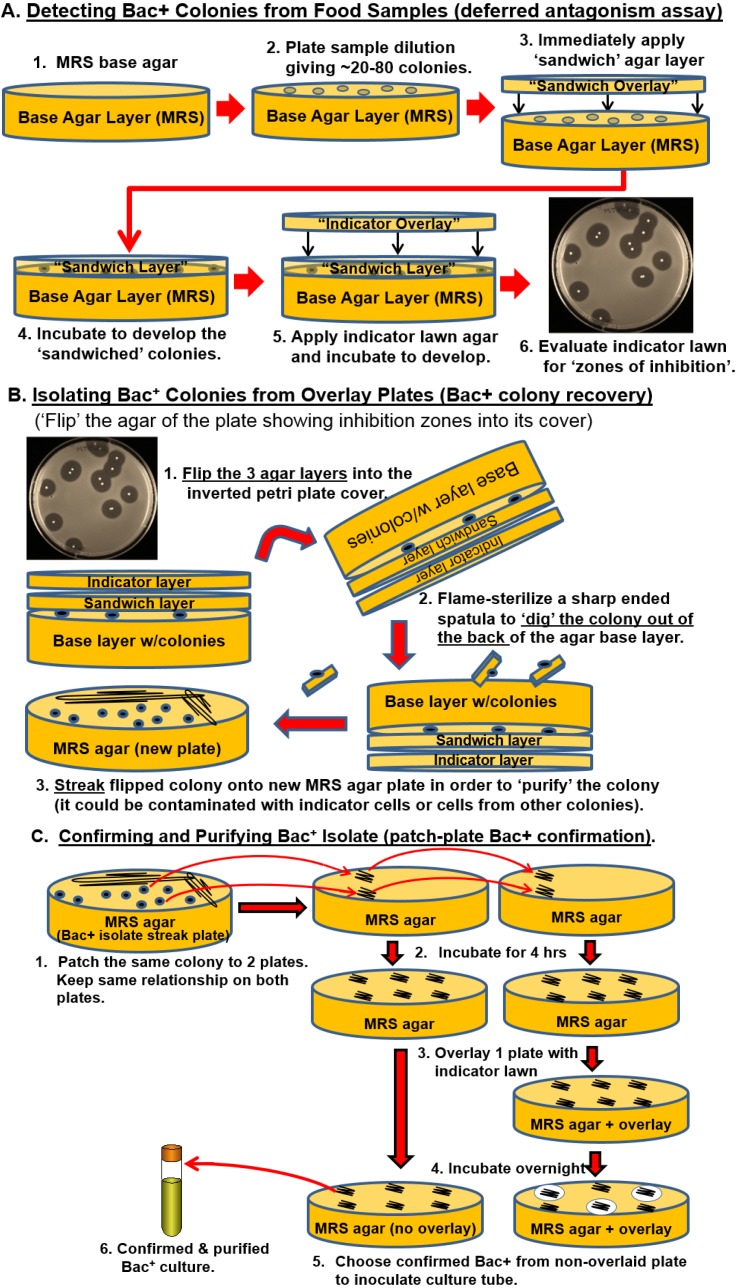
Various protocols used in this study. Panel **A**, colony overlay assay (deferred antagonism) to identify Bac^+^ colonies by the sandwich-overlay technique. Panel **B**, isolation of Bac^+^ colonies from sandwich overlay plates. Panel **C**, the double patch plate method for purification and confirmation of Bac^+^ colonies and generation of the stock culture.

Food items were obtained from 4 local supermarkets in Stillwater, OK. These consisted of raw meat, fruit, vegetables, and herbs. In addition, beef cattle rumen, raw milk, and fecal samples were obtained from animals maintained by the Dept. of Animal Science, Oklahoma State University (Stillwater, OK, USA). Enrichment was performed in Whirl-Pak^®^ filter bags (Nasco, Fort Atkinson, WI, USA) as a 10-fold dilution in MRS Broth (Difco) for 24 h (or 4 h) at 30 °C. Enriched broth was serially diluted and spread plated onto buffered (pH 7.4 with 0.1 M sodium phosphate dibasic and 8 mM sodium phosphate monobasic) MRS agar plates (Figure 1). Plated samples were immediately covered with MRS agar (the sandwich layer) and incubated at 37 °C for 12 to 48 h. The sandwiched colonies were then overlaid with tempered molten “soft” MRS agar (0.75% agar) inoculated with 1 mL of indicator organism (e.g., *Listeria monocytogenes*, ~1 × 10^9^ cfu/mL) per 100 mL of molten media and again incubated at 37 °C until the indicator lawn grew to completion. Bacteriocin-producing colonies were then isolated as described previously [9] and processed as depicted in Figure 1.

### 2.3. Exclusion of Other Inhibitors

The possibility that other inhibitors common to LAB may give inhibitory reactions appearing as bacteriocin activity were eliminated using the following assays [10,11,12].

Bacteriophage may demonstrate zones of inhibition when concentrated phage-containing preparations are spotted on a susceptible host (*i.e.*, “confluent lysis”). When the bacteriophage-containing suspension is titered to extinction, individual plaques would be observed if inhibition zones were due to phage infection.

Hydrogen peroxide may be produced by some LAB and can be inhibitory to organisms (although *L. monocytogenes* is catalase-positive). The possibility of hydrogen peroxide activity was eliminated by addition of catalase as described previously, including control solutions of 3% hydrogen peroxide [13].

Acid inhibition was eliminated from consideration by neutralizing spent culture supernatant fractions and/or buffering base agar/sandwich overlays with phosphate buffer during bacteriocin isolation and bacteriocin confirmation procedures. Control assays included cell-free culture supernatants from a bacteriocin-negative (Bac^−^) lactic acid-producing culture, *Lb. delbrueckii* 4797.

The application of protease to hydrolyze the protein content of bacteriocins and eliminate activity was examined by adding 20 μL (*i.e.*, 21 IU) of pronase E (1060 units/mL; Sigma cat. No. 42H0749) to 80 µL of bacteriocin preparation, incubated at 30 °C for 1 h, and then spotted on indicator lawns to view presence/absence of bacteriocin activity. Control reactions used water instead of protease.

### 2.4. Extraction of Total Bacterial DNA

Prior to extraction of DNA, the Bac^+^ isolates were inoculated into MRS broth and incubated at 30 °C for 16 h. DNA was recovered from cell pellets of 1-mL of cells by the bead-beating and ice-shaking procedure of Coton and Coton [14] and then stored at −20 °C until needed. The concentration of DNA was measured using a NanoDrop^®^ ND-1000 spectrophotometer (Thermo Scientific, Wilmington, DE, USA).

### 2.5. Identification of Isolates by 16S rRNA Gene Amplification, Sequencing, and Analysis

The identification of Bac^+^ bacteria was determined using PCR amplification with universal 16S ribosomal RNA primers 515F (5’-GTGCCAGCMGCCGCGGTAA-3’) and 1391R (5’-GACGGGCGGTGTGTRCA-3’) as described by Turner *et al.* [15]. Thermal cycling was performed using a PTC-200 Peltier Thermal Cycler (MJ Research/Bio-Rad Laboratories, Hercules, CA, USA) as described previously [16]: initial denaturation at 95 °C for 4 min, followed by 30 cycles of 94 °C for 1 min (denaturation), 60 °C for 45 s (annealing), 72 °C for 1 min (extension), followed by a final extension cycle at 72 °C for 4 min, and a final hold at 4 °C. Amplimers of 16S rRNA genes were purified using the GenCatch™ Advanced PCR Extraction Kit (Epoch Life Sciences, Missouri City, TX, USA) according to the manufacturer’s recommendations and eluted DNA was stored at −20 °C until needed. Purified DNA was submitted to the Dept. of Biochemistry and Molecular Biology ”DNA Core Facility” (Oklahoma State University) for sequencing using an automated DNA sequencer via “BigDye™”(Life Technologies, Grand Island, NY, USA)-terminated reactions analyzed on an ABI Model 3700 DNA Analyzer. ABI sequence files were analyzed using MEGA5 (The Biodesign Institute, Tempe, AZ, USA) [17] by cutting out 5′ and 3′ regions of high background noise. Consensus sequences were identified using NCBI’s Nucleotide BLAST and then compiled together and aligned to create a maximum likelihood phylogenetic tree using MEGA 6 [18].

## 3. Results

### 3.1. Isolation of Bac^+^ Strains of LAB

In this study, our objectives were to identify bacteriocinogenic LAB indigenous to retail foods and animal sources that were inhibitory to *L. monocytogenes*. Bac^+^ bacteria were isolated from 23 of 170 retail food samples (14% occurrence) and from 11 of 110 (10%) animal-related sources (Table 1). Inhibitory activity was deemed to be the result of bacteriocin activity as there was no evidence of inhibition against *L. monocytogenes* due to bacteriophage, acid, or hydrogen peroxide, and protease treatment effectively demonstrated the protein nature of the active agent by hydrolysis of bacteriocin proteins that resulted in the subsequent loss of activity.

**Table 1:** Bacteriocin-producing strains isolated or used in this study.

Organism	Strain	Source/Reference
*Enterococcus faecium*	326F	This study; bovine fecal sample
*Enterococcus faecium*	FS97-2	Vegetable [9]
*Enterococcus faecium*	JCP B-5	This study; pork sausage
*Enterococcus faecium*	JCP M-2	This study; pork sausage
*Enterococcus faecium*	Thyme 2	This study; thyme
*Enterococcus faecium*	Thyme 3	This study; thyme
*Enterococcus faecium*	Milk 5	This study; raw milk
*Enterococcus faecium*	JCP-9	This study; pork sausage
*Enterococcus faecium*	Milk 12	This study; raw milk
*Enterococcus faecium*	Pop 4	This study; dog feces
*Enterococcus faecium*	FS56-1	Mushrooms [9]
*Enterococcus durans*	FS707	This study; bovine fecal sample
*Enterococcus hirae*	323F	This study; bovine fecal sample
*Enterococcus hirae*	323 RL1	This study; bovine rumen fluid
*Enterococcus hirae*	341 FA	This study; bovine fecal sample
*Enterococcus thailandicus*	RP-1	Raw pork [5]
*Enterococcus thailandicus*	FS92	Raw pork [9]
*Enterococcus faecalis*	BJ-12	Muriana culture collection
*Enterococcus faecalis*	BJ-13	Muriana culture collection
*Enterococcus faecalis*	BJ-27	Muriana culture collection
*Carnobacterium maltaromaticum*	LGBF-1	This study; ground beef
*Carnobacterium maltaromaticum*	GBF-1	This study; ground beef
*Carnobacterium maltaromaticum*	COG-1	This study; collard greens
*Carnobacterium maltaromaticum*	COG-2	This study; collard greens
*Carnobacterium maltaromaticum*	CHW-1	This study; chicken wings
*Carnobacterium maltaromaticum*	TOF-1	This study; tofu
*Carnobacterium maltaromaticum*	GPK-1	This study; ground pork
*Carnobacterium maltaromaticum*	GAC-1	This study; ground chuck
*Carnobacterium maltaromaticum*	GAC-2	This study; ground chuck
*Pediococcus acidilactici*	Bac3	Ground turkey [5]
*Pediococcus acidilactici*	FS707 S4	This study; bovine fecal sample
*Lactobacillus ingluviei*	FS60	Cheese [9]
*Lactobacillus curvatus*	BJ-5	Muriana culture collection
*Lactobacillus curvatus*	BJ-18	Muriana culture collection
*Lactobacillus curvatus*	BJ-21	Muriana culture collection
*Lactobacillus curvatus*	BEEF 2L-1	This study; ground beef
*Lactobacillus curvatus*	BEEF 3	This study; ground beef
*Lactobacillus curvatus*	FS36-1	Ground beef [9]
*Lactobacillus curvatus*	FS44-B	Ground pork [9]
*Lactobacillus curvatus*	FS47	Ground beef [9]
*Lactobacillus curvatus*	FS80-2	Muriana culture collection
*Lactobacillus sakei*	FS707 S1	This study; bovine fecal sample
*Leuconostoc mesenteroides*	BFS-1	This study; breakfast sausage
*Staphylococcus gallolyticus*	707RS	This study; bovine rumen fluid
*Staphylococcus gallolyticus*	341RL	This study; bovine rumen fluid
*Lactococcus lactis*	BJ-23	Muriana culture collection
*Lactococcus lactis*	FL-1	This study; lettuce (foxy)
*Lactococcus lactis*	FL S-1	This study; lettuce (foxy)
*Lactococcus lactis*	FS91-1	Fruit [9]
*Lactococcus lactis*	FS95	Vegetables [9]
*Lactococcus lactis*	FS162	Muriana culture collection
*Lactococcus lactis*	ASPG-1	This study; asparagus
*Lactococcus lactis*	PJP-1	This study; peppers
*Lactococcus lactis*	RDSH-1	This study; radish (red)
*Lactococcus lactis*	SL-1	This study; shredded lettuce
*Lactococcus lactis*	YO-1	This study; yellow onion
*Lactococcus lactis*	SP-1	This study; sweet potato
*Lactococcus lactis*	RD	This study; radish
*Lactococcus lactis*	GBN-1	This study; green beans
*Lactococcus lactis*	BSP	This study; bean sprouts
*Serratia plymuthica*	POT-1	This study; Russet Potato
*Serratia ficaria*	CCEL-1	This study; Chinese celery

*Enterococcus* spp. represented the largest group of organisms and were often associated with animal-related sources, and *En. faecium* along with *Lactococcus lactis* comprised our largest phylogenetic groups (Table 1). *Lactococcus lactis* were obtained from the largest variety of sources, including green beans, radish, sweet potato, yellow onion, shredded lettuce, jalapeno peppers, asparagus, whole lettuce, and bean sprouts (Table 1). *Lactobacillus curvatus* isolates were obtained only from ground beef and, as psychrotrophs, they can grow well at refrigeration temperatures leading to shortened shelf life and spoilage. The sole *Leuconostoc mesenteroides* isolate was obtained from breakfast sausage. *Carnobacterium maltaromaticum* isolates were obtained from ground beef, collard greens, chicken wings, tofu, ground pork, and ground Angus chuck. *Enterococcus faecium* isolates were obtained from thyme herbs and pork sausage. The *Serratia plymuthica* isolate was obtained from russet potatoes while the *Serratia ficaria* isolate was obtained from Chinese celery. Although 25 samples were tested, no Bac^+^ LAB were isolated from fresh fruit in the current study.

### 3.2. Differentiation of Bacterial Isolates

Newly-isolated Bac^+^ bacteria spanned 6 different bacterial genera of lactic acid bacteria including *Lactococcus*, *Carnobacterium*, *Enterococcus*, *Lactobacillus*, *Leuconostoc*, and *Pediococcus* (Table 1). Similar Bac^+^ bacteria from the same food sample were differentiated from each other by some type of distinguishing characteristics in order to minimize repeat isolations of the same organism. One method of differentiating Bac^+^ strains is by inhibitory patterns against themselves; Bac^+^ strains are not inhibitory to themselves because of bacteriocin immunity genes. For instance, *Carnobacterium maltaromaticum* COG-1 (indicator) was inhibited by *C. maltaromaticum* COG-2 (spotted culture), but not inhibited by its own spotting, proving that they are two different strains. This type of result only proves anything if one strain inhibits the other, or both inhibit each other, but provides no conclusive answer if neither inhibits the other as they could either be the same strain or conversely, simply insensitive to each other’s bacteriocin. In the latter case, differences of inhibitory spectra involving other strains may be used to distinguish Bac^+^ strains as was observed with *Lb. curvatus* Beef 2L-1 and Beef 3 whereby Beef 2L-1 was inhibited by *En. faecalis* BJ-13 but Beef 3 was not. Other differentiations were based on sequence analysis of bacteriocin structural gene sequences. For instance, *En. faecium* Thyme 2 possessed one enterocin structural gene (*ent*A) while *En. faecium* Thyme 3 possessed two (*ent*A, *ent*B); similar differences were observed for *En. hirae* 343F (*ent*A, *mr10*AB, *mun*A) *vs.* *En. hirae* 323 RL1 (*ent*A, *mr10*AB), and *En. faecalis* BJ-12 (*ent*A), *En. faecalis* BJ-13 (*bac*A), and *En. faecalis* BJ-27 as described recently by Henning *et al.* [16].

### 3.3. Inhibition of Foodborne Pathogens

**Figure 2:**
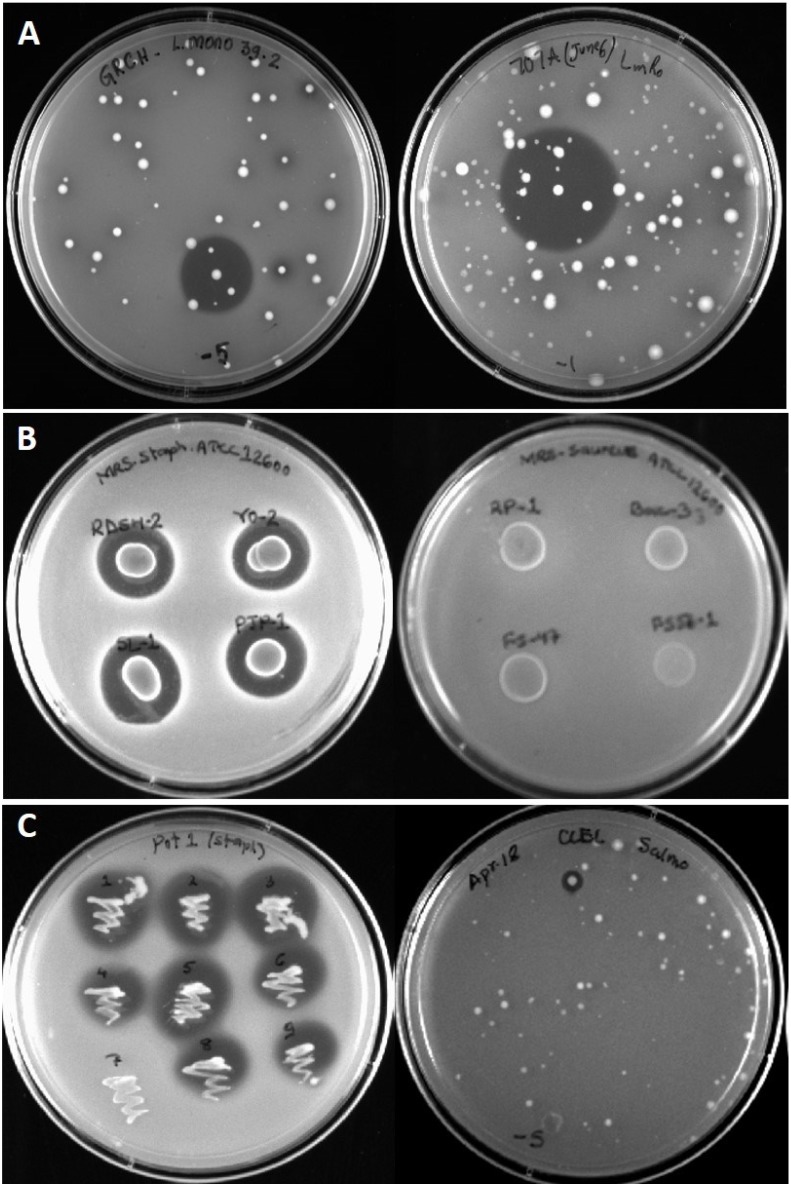
Bacteriocin inhibition zones from overlaid producer colonies, deferred antagonism spots, or patch-plate isolations. Panel **A**, representative bacteriocin inhibition zones obtained with “sandwich overlay” plates of food enrichment samples overlaid with *Listeria monocytogenes* 39-2 indicator lawns. Panel **B**, deferred antagonism assay of culture spots overlaid with *Staphylococcus aureus* ATCC 12600. Panel **C**, patch plate of *Serratia plymuthica* POT-1 (left) overlaid with *S. aureus* ATCC 12600 and an inhibition zone from *Serratia ficaria* CCEL-1 (right) against *Salmonella* Typhimurium H3380 used as a primary indicator screen.

The predominant primary indicator organism during this study was *L. monocytogenes* and an abundant supply of Bac^+^ LAB capable of inhibiting *L. monocytogenes* were isolated (Table 1; Figure 2A). Other organisms were also used as “secondary indicators” once the Bac^+^ isolates were obtained against *L. monocytogenes*. Although this approach was met with limited success, we were able to find some isolates capable of inhibiting *Staphylococcus aureus* ATCC 12600 (Figure 2B). However, no isolates were found that were inhibitory to *E. coli* O157:H7 or *Salmonella* Typhimurium using this secondary approach. A few food samples were tested directly with *Salmonella* as a primary indicator screen leading to the isolation of *Serratia ficaria* CCEL-1 that inhibited *Salmonella* Typhimurium (Figure 2C). When the *Serratia* strains were tested on other organisms, they were also found to inhibit *S. aureus* ATCC 12600 (Figure 2C), *En. faecalis*, and *L. monocytogenes* (Table 2).

**Table 2:** Inhibitory activity elicited by select Bac^+^ strains against various foodborne pathogens.

Select Bacteriocin-Producing Strains	*Listeria monocytogenes* 39-2	*Staphylococcus aureus* ATCC 12600	*Staphylococcus aureus* ISP 178	*Enterococcus faecalis* ATCC 19433
*Enterococcus thailandicus* RP-1	++ ^1^	-	-	ND ^2^
*Enterococcus faecium* FS56-1	++++	-	-	ND
*Enterococcus durans* FS707	++++	-	-	++
*Lactobacillus curvatus* FS47	++++	-	-	-
*Lactobacillus curvatus* Beef3	++++	-	-	-
*Lactobacillus curvatus* FS44-B	+++	-	-	-
*Lactococcus lactis* BSP	+++	++	++	+
*Lactococcus lactis* SL1	++	+++	+++	+
*Lactococcus lactis* RDSH-1	+++	+++	+++	+
*Lactococcus lactis* ASPG-1	+++	+++	+++	+
*Lactococcus lactis* YO-1	+++	+++	+++	+
*Lactococcus lactis* FLS1	+	+	+	+
*Lactococcus lactis* GBN-1	++	+	+	+
*Lactococcus lactis* PJP-1	+++	+++	+++	+
*Pediococcus acidilactici* Bac3	++++	-	-	ND
*Serratia plymuthica* POT-1 ^3^	++	++	++	+
*Serratia ficaria* CCEL-1 ^3^	+	+	+	+

^1^ Inhibitory reaction designations are based on degree of inhibitory activity observed: no inhibition (-), slight inhibition (+), moderate inhibition (++, +++), and highest degree of inhibition (++++); ^2^ ND, not determined; ^3^ Inhibition was also observed against *Salmonella* Typhimurium (Figure 2).

### 3.4. Phylogenetic Analysis

**Figure 3:**
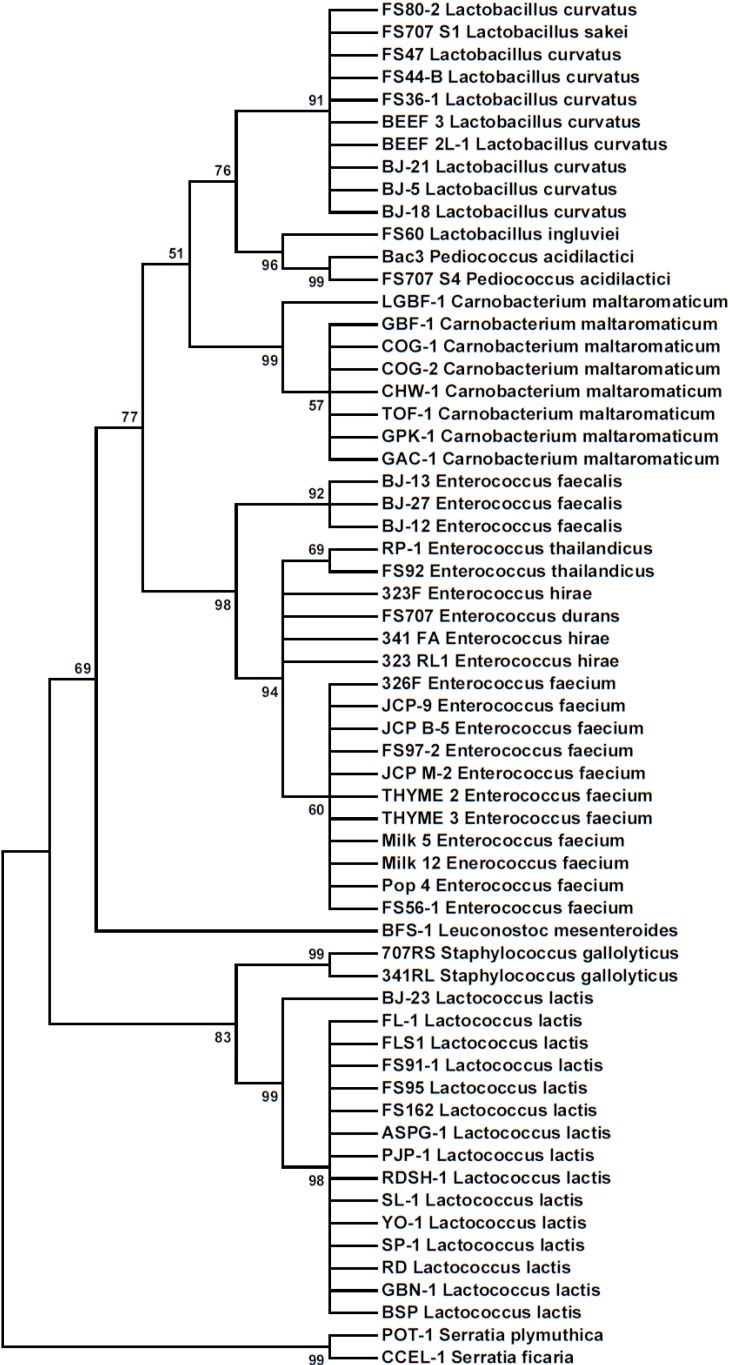
Phylogenetic tree of 16S rRNA partial sequences from Bac**^+^** bacteria by the Maximum Likelihood method. The phylogenetic relationship was inferred using the Tamura-Nei model [21] showing the tree with the highest log likelihood (−2162.4232). The percentage of trees in which the associated taxa clustered together is shown next to the branches.

Bacteria were identified by DNA sequence analysis of amplimers obtained from PCR reactions performed with universal primers for 16S rRNA genes. Some Bac^+^ strains were included for 16S rRNA identification that were isolated previously and identified incorrectly by phenotypic methods [9] or API 50 CH panels [5], demonstrating the accuracy of this method of identification over phenotypic methods. For instance, *En. faecium* FS56-1 and FS97-2 were incorrectly identified as *Lactococcus lactis* using a variety of phenotypic characteristics [9] and later, along with *En. thailandicus* FS92 and RP-1, were again incorrectly confirmed as *Lc. lactis* using API 50CH panels [5]. The phylogenetic distribution of Bac^+^ organisms based on 16S rRNA partial sequences from this study are shown in Figure 3 as a Maximum Likelihood Tree of sequence relatedness constructed from 61 nucleotide sequences using the MEGA 6 genetic analysis software.

## 4. Discussion

The analysis of Bac^+^ bacteria from common market foods and samples from animal sources in this study implicates a variety of bacterial genera with the ability to produce bacteriocins including some genera that are not members of the lactic acid bacteria. The actual prevalence of Bac^+^ bacterial strains in retail foods are likely much higher than what we have identified because our findings represent only those that were inhibitory to our primary indicator strain. Select indicator strains may not be susceptible to all Bac^+^ organisms, some of which may show up as Bac^−^ against “the wrong” given indicator organism. The burden of using all the indicator organisms in this study as primary indicator screens prevented as thorough an isolation rate as was obtained with *L. monocytogenes*, and retesting Bac^+^ isolates against secondary indicator organisms has its limitations. Also, the use of MRS broth/agar media limits the detection to those organisms that can grow on it, providing a selective preference (in this study) for lactic acid bacteria.

Several modifications were accommodated in this study over prior studies; notably all isolates were subjected to 16S rRNA sequencing and identification. This provided more accurate sequence-based identification and demonstrated that some previously isolated strains were incorrectly identified as lactococci using less stringent identification methods. Similar results have been observed in other comparisons of 16S rRNA sequence-based identification compared to phenotypic test kits [19,20].

Enrichment schemes were also altered from 24 to 4-h enrichments when we questioned whether lactococci that are known to grow to 10^10^ cfu/mL in culture media may predominate in enriched food samples over other Bac^+^ bacteria that may not grow to similarly high levels, given that the screening process is based on plating schemes examining higher end dilutions. Due to the high prevalence of *Lactococcus lactis*, we reduced enrichment times to 4 h to provide an opportunity to isolate other organisms that may not grow to the level of *Lactococcus*. Also, the use of buffered agar media during isolation of Bac^+^ LAB against acid-sensitive pathogenic indicator organisms eliminated the likelihood of false-positives from inhibition zones due to acid inhibition.

In prior studies, a sensitive lactic acid indicator organism (*i.e.*, *Lb. delbrueckii* 4797) was often used as a primary indicator of Bac^+^ bacteria that were then used to test on organisms of interest (*i.e.*, *L. monocytogenes*). This approach was stopped because of the possibility that the initial isolation may have restricted the spectrum of activity because the initial selection was limited to that showing inhibition towards the LAB (Figure 2B). In this study, *L. monocytogenes* was used directly as a primary indicator strain and numerous isolated were obtained that were inhibitory to *Listeria*. However, a similar approach using other organisms (*i.e.*, *Salmonella*, *E. coli* O157:H7, *etc.*) as primary indicator organisms would similarly benefit from improved detection frequencies than to examine them by a secondary screening regimen. This is evidenced by the detection of an isolate inhibitory to *Salmonella* when *Salmonella* was used as a primary susceptible indicator organism to directly screen for inhibitory colonies (Figure 2C). Despite the use of various additional organisms via a secondary screening approach, we did identify inhibition to other pathogens (Table 2) that may well suit our interest in examining bacteriocins that may have different modes-of-action with the purpose of combining them in food applications.

## 5. Conclusions

This work extends our knowledge of the ubiquitous distribution of Bac^+^ bacteria in foods and provides further argument that consumers may be ingesting Bac^+^ bacteria or their byproducts on foods they already eat. Despite the heavy emphasis on food safety in the past decade whereby antimicrobials are added/sprayed onto raw or processed meats, vegetables, and produce for the interest of “pathogen reduction”, there does not seem to be a scarcity of Bac^+^ bacteria on foods, providing researchers a broad array of antimicrobials for potential use as food preservatives. The organisms and bacteriocins described in this work are currently being examined in greater detail to accommodate their application as antimicrobials against *L. monocytogenes* in food applications.
